# Stabilization of the Dimeric Birch Pollen Allergen Bet v 1 Impacts Its Immunological Properties*

**DOI:** 10.1074/jbc.M113.518795

**Published:** 2013-11-05

**Authors:** Stefan Kofler, Chloé Ackaert, Martin Samonig, Claudia Asam, Peter Briza, Jutta Horejs-Hoeck, Chiara Cabrele, Fatima Ferreira, Albert Duschl, Christian Huber, Hans Brandstetter

**Affiliations:** From the ‡ Structural Biology Group, Department of Molecular Biology,; the § Division of Allergy and Immunology, Department of Molecular Biology, and; the ¶ Division of Chemistry and Bioanalytics, Department of Molecular Biology, University of Salzburg, 5020 Salzburg, Austria

**Keywords:** Allergen, Crystal Structure, Mass Spectrometry (MS), Post-translational Modification, Protein Assembly, Dimerization, Noncanonical Amino Acid Incorporation, Polysulfide Linking, Position-specific Alteration of Genetic Code

## Abstract

Many allergens share several biophysical characteristics, including the capability to undergo oligomerization. The dimerization mechanism in Bet v 1 and its allergenic properties are so far poorly understood. Here, we report crystal structures of dimeric Bet v 1, revealing a noncanonical incorporation of cysteine at position 5 instead of genetically encoded tyrosine. Cysteine polysulfide bridging stabilized different dimeric assemblies, depending on the polysulfide linker length. These dimers represent quaternary arrangements that are frequently observed in related proteins, reflecting their prevalence in unmodified Bet v 1. These conclusions were corroborated by characteristic immunologic properties of monomeric and dimeric allergen variants. Hereby, residue 5 could be identified as an allergenic hot spot in Bet v 1. The presented results refine fundamental principles in protein chemistry and emphasize the importance of protein modifications in understanding the molecular basis of allergenicity.

## Teaser

**Background:** Frequently reported dimerization of allergens may contribute to their allergenicity.

**Results:** Polysulfide-bridged allergen dimers exhibit different allergenic properties compared with the monomer.

**Conclusion:** The N-terminal region has a distinct susceptibility for modifications and impacts its protein-protein interaction characteristics.

**Significance:** The crystal structures well mimic transient dimerization of the allergens in solution, providing a rational for effective IgE cross-linking on effector cells.

## Introduction

Allergy is a steadily increasing health problem, with ∼25% of the population in Western countries affected. More than 800 allergens have been identified and classified (1), with three-dimensional structures determined for many of them. There are several biophysical properties shared by allergens, *e.g.*, their small size, high stability, and solubility or foreignness to the affected host. Further, protein features like enzymatic activity or glycosylation patterns could be identified as common allergen-related properties (2, 3). However, despite this enormous increase of biological knowledge, the understanding of the molecular mechanisms constituting the allergenicity of some proteins remains so far elusive.

One relevant but poorly understood feature of allergens is the capability of undergoing conformational changes, especially dimerization. Many important allergens are reported to form homodimers or -oligomers, *e.g.*, Phl p 7 from timothy grass pollen (4), Equ c 1 from horse (5), or Ara h 1 from peanut (6). The structure of dimeric Bos d 5 from bovine milk was published in complex with two IgE Fab fragments (7). This provides a model for effective IgE cross-linking on effector cells *in vivo*, a hallmark event in the manifestation of allergic disease. Another interesting case of allergen dimerization is reported for Bet v 4 from birch pollen and Phl p 7. For these allergenic polcalcins, dimerization was shown to be temperature- and time-dependent and facilitated by domain swapping (8). The dust mite allergen Der p 1, a cysteine protease, occurs as a functional dimer. Protease inhibition results in dissociation of the dimer and a reduced allergenic activity (9). In a general examination of transient dimerization of allergens, 55 allergen crystal structures were analyzed, showing more than 80% of them crystallizing as symmetric dimers or oligomers (10).

Partly conflicting data on the allergenic significance of dimerization were reported for the birch pollen allergen Bet v 1, an outstandingly well characterized model system in immunology. It is the archetypical representative of the pathogenesis related protein family 10. Bet v 1 occurs in several natural isoforms with high sequence similarity, yet drastically differing immunogenic and allergenic properties (11). One study describes correlation of Bet v 1.0401 dimerization with protective IgG and IgA titers (12). In contrast, dimerization of the hyperallergenic Bet v 1.0101 (further referred to as Bet v 1a) was shown to be an essential feature for its IgE cross-linking ability and therefore is crucial for its allergenicity (13). Changes in the oligomeric state of Bet v 1a were repeatedly reported (11, 13–16).

In the current study we set out to elucidate the mechanism and functional relevance of dimerization of Bet v 1a by combining crystallographic, mass spectrometric, and immunological methods. These results identified residue 5 of Bet v 1a as an allergenic hot spot.

## EXPERIMENTAL PROCEDURES

### 

#### 

##### Protein Preparation

Recombinant Bet v 1 (WT and mutants) was expressed in *Escherichia coli* strain BL21(DE3), using a modified pET-28b vector, lacking the N-terminal His_6_ tag. Cells were grown in 600 ml of LB medium supplemented with 20 μg/ml kanamycin at 37 °C to an *A*_600_ of 1.0. After adding 1 mm isopropyl β-d-thiogalactopyranoside, expression was performed for 4 h at 37 °C. Proteins were purified in principle as described elsewhere (17), with some minor changes of the protocol. As final step, multiple size exclusion chromatography (Superdex 75) was used to separate monomeric and dimeric forms.

##### Characterization of Dimeric Bet v 1a

Analysis of the dimeric state of Bet v 1 was monitored by SDS-PAGE under reducing (containing β-mercaptoethanol) and nonreducing conditions. For clear native PAGE, 15% polyacrylamide gels were produced according to SDS-PAGE, just omitting SDS. As loading dye, 10% glycerol was used. Anode buffer contained 25 mm imidazole, pH 7.0, cathode buffer 7.5 mm imidazole, pH 7.0, and 50 mm Tricine. For Western blot, polyclonal rabbit anti-rBet v 1a IgG antibodies (affinity-purified with recombinant protein G; Pierce) were used. Rabbit IgG was detected with alkaline phosphatase-conjugated goat anti-rabbit IgG Fc (Jackson ImmunoResearch, West Grove, PA).

##### Crystallization of Dimeric Bet v 1

Bet v 1a crystals were obtained by sitting drop vapor diffusion, using 5 mg/ml protein concentration in 20 mm imidazole, pH 7.4, and 50 mm NaCl. The crystallization buffer was composed of 0.1 m sodium acetate trihydrate, pH 5.5, and 29% polyethylene glycol 3350 as precipitants. Both crystal species (space groups P2_1_ and C2) grew in the same conditions. For reduction, crystals obtained from linked Bet v 1a Y5C dimer were transferred in a drop of mother liquor containing 1% β-mercaptoethanol and incubated for several seconds.

##### Data Collection and Structure Determination

Crystals were flash frozen in a stream of nitrogen gas at 100 K. X-ray diffraction data sets were collected in a single pass at Beamline ID29 at the European Synchrotron Radiation Facility (Grenoble, France). Diffraction data were indexed, scaled, and further processed using CCP4 software suite. All structures were solved by molecular replacement, using Phaser. As search model, Bet v 1a (Protein Data Bank accession code 4A88 (18)) was used. Refinement was performed in Refmac5 using an anisotropic B-factor model (19) and monitored throughout using an *R*_free_ calculated with 5% of the unique reflections. Conservative restraints were used to account for the dominant scattering contribution of the polysulfide linker. Model building was done in COOT (20). All figures were generated using PyMOL (21). The quality of all models was checked using MolProbity (22) and NQ-Flipper (23). Coordinates have been deposited with accession codes 4BK6, 4BK7, 4BKC, and 4BKD.

##### Mass Spectrometry Analysis of Dimeric “WT Bet v 1a” and Genetic Mutants Bet v 1a Y5C/Y5F

Proteins were reduced, alkylated, and digested using the ProteoExtract all-in-one trypsin digestion kit (Calbiochem). Resulting peptides were separated by reverse phase HPLC directly coupled to an electrospray ionization quadrupole TOF mass spectrometer (Q-Tof Ultima Global; Waters/Micromass). After washing the trap column with 0.1% (v/v) formic acid, peptides were eluted with an acetonitrile gradient (Solvent A: 0.1% (v/v) formic acid, 5% (v/v) acetonitrile; solvent B: 0.1% (v/v) formic acid, 95% (v/v) acetonitrile; 5–45% B in 90 min) at flow rate of 300 nl/min. For ionization, the Waters Nanoflow spray head was used. Singly, doubly, triply, and quadruply charged ions were selected for fragmentation by collision with argon. Data acquisition and instrument control were done with the MassLynx software V4.1 (Waters). The instrument was calibrated with the fragment ions of [Glu^1^]Fibrinopeptide B (Sigma). Survey and fragment spectra were analyzed using ProteinLynx Global Server 2.2.5 (Waters) with automatic data validation. For sequence identification, an in-house database containing the Bet v 1a sequence were used. Deviations from the Bet v 1a sequence were identified both by *de novo* sequencing and the modification/substitution search program of ProteinLynx.

##### HPLC-MS Analysis of the Linker between Cys^5^ (A) and Cys^5^ (B)

Monolithic 150 × 0.20-mm inner diameter polystyrene divinylbenzene capillary columns were prepared according to a previously published protocol (24). Separations were performed with a capillary HPLC system (model UltiMate3000; Dionex Benelux) including a detector equipped with a 3-nl Z-shaped capillary detection cell. Separations were generally accomplished at 55 °C with gradients of acetonitrile in 0.050% aqueous trifluoroacetic acid at a flow rate of 1 μl/min. MS analysis was performed with a linear ion trap-Orbitrap mass spectrometer (model LTQ XL; ThermoFisher Scientific), essentially under optimized conditions as published previously (25). A nanoelectrospray ionization source was utilized with a 20-μm inner diameter fused silica capillary and a tip drawn to 10-μm inner diameter (New Objective, Woburn, MA). The instrument was operated in positive electrospray ionization mode with a spray voltage of 1.45 kV, a capillary voltage of 41.0 V, a capillary temperature of 250 °C, a tube lens voltage of 155.0 V, and an Orbitrap target value of 10^6^. The MS parameters were optimized in the range of *m*/*z* 440–2,500 by infusing a solution of myoglobin in water-acetonitrile (80:20) containing 0.05% triflouroacetic acid at a concentration of 1.3 pmol/μl at resolutions of 7,500–100,000 at *m*/*z* 400. For MS/MS experiments, a data-dependent precursor selection method was used, and the fragmentation was performed in the linear ion trap with collision induced dissociation at 35% normalized collision energy. Mass calibration was accomplished with the commercially available positive calibration solution for LTQ XL and LTQ hybrids (Sigma Aldrich). The mass spectra were analyzed by using the data evaluation software Xcalibur (Thermo Scientific) and the implemented deconvolution tool Xtract.

##### Suppression/Induction of Bet v 1a Y5C Dimerization in the Dialysis Tubing

For all approaches, Spectra/Por3 dialysis membrane with a molecular mass cutoff of 3,500 Da was used. The Bet v 1a Y5C sample was prepared according to the purification protocol up to and including hydrophobic interaction chromatography. Dialysis was performed against 20 mm imidazole, pH 7.4, overnight. Dialysis tubings were pretreated by boiling 10–15 times, always using fresh distilled H_2_O. Additives (EDTA, CuCl_2_, NiCl_2_, and FeCl_2_) were added to the dialysis buffer. Elemental sulfur was added directly to the sample in the dialysis tubings.

##### Induction of Bet v 1a Y5C Dimerization in the Eppendorf Tube

The same protein sample was used as for dialysis approaches. As a first step, the sample was rebuffered using gel filtration, to bring the protein in a suitable buffer for cysteine oxidation (25 mm HEPES, pH 7.5). Solid sulfur and/or metals were added directly to the sample and were incubated on the rotator.

##### Database Similarity Search

Database search was performed using TopSearch from the COPS server for Protein Structure Analysis (compare Ref. 26).

##### 1-Anilino-8-naphthalene sulfonate Displacement Assay

50 μl of protein solution (5 and 10 μm final concentration) were mixed with deoxycholate (DXC) in a 96-well UV-Star plate in different molar ratios. Mixtures were incubated overnight at 4 °C. Prior to the measurements, 50 μl of ANS^2^ (50 μm final concentration) were added, and the mixtures were incubated for another 5 min at room temperature. ANS was excited at 350 nm, and the resulting fluorescence signal was measured at 486 nm.

##### Modification of Bet v 1a Y5C with Glutathione

Bet v 1a Y5C was incubated with a mixture of reduced glutathione:oxidized glutathione disulfide (ratio 1:10) overnight at 4 °C to covalently modify Y5C with the glutathione tripeptide. Monomeric mixed disulfide Bet v 1 was separated from unmodified dimeric Bet v 1 by gel filtration.

##### Mediator Release Assays

The allergenic potential was assessed by rat basophile degranulation assays performed as previously described (27). In short, rat basophile leukemia 2H3 cells were transfected with the human high affinity IgE receptor (FcϵRI) and were passively sensitized with serum IgE from birch pollen allergic donors. Antigen-dependent β-hexosaminidase release into the supernatant was measured by enzymatic cleavage of the fluorogenic substrate 4-methyl umbelliferyl-*N*-acetyl-β-glucosaminide and expressed as a percentage of total enzyme activity of Triton X-100-treated cells.

##### ELISA Experiments

Maxisorp plates (NalgeNunc, Rochester, NY) were coated with monoclonal mouse anti Bet v 1 antibody BIP-1 (28) (200 ng/well in 50 μl of PBS) overnight at 4 °C. Wells were blocked with TBS, pH 7.4, 0.05% (v/v) Tween, 0.5% (w/v) BSA and incubated with serial dilutions of proteins, starting with 0.1 μg/ml, overnight at 4 °C. Plates were incubated with patients' sera diluted 1:10 overnight at 4 °C. Detection of bound IgE was performed with alkaline phosphatase-conjugated monoclonal anti-human IgE antibodies (BD Biosciences, Franklin Lakes, NJ), after incubation for 1 h at 37 °C and for 1 h at 4 °C. 10 mm 4-nitrophenyl phosphate (Sigma-Aldrich) was used as substrate, and *A* was measured at 405/492 nm.

##### Stimulation of Primary Dendritic Cells and Cytokine Analysis

Primary dendritic cells were isolated from buffy coats obtained from healthy donors (IL-12, 16 donors used; TNF-α, 19; IL-6, 20; MCP-1, 17; TARC, 13; MDC, 6; provided from the blood bank in Salzburg) using the MACS BDCA1+ kit (Miltenyi Biotech, Bergisch Gladbach, Germany). Cells were plated out in primary dendritic cell medium and stimulated with 50 μg/ml protein, 10 ng/ml lipopolysaccharide, and 30 ng/ml thymic stromal lymphopoietin as controls. After 24 h, the supernatant was used for the analysis of secreted cytokines by mean of ELISA (kits used from PeproTech (Rocky Hill, NJ) and R&D Systems (Minneapolis, MN)).

## RESULTS

### 

#### 

##### Identification of the Recombinant Bet v 1 Dimer

**FIGURE 1.**
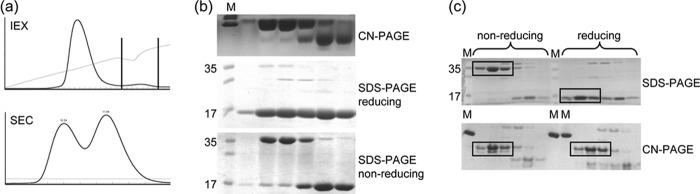
**Identification of dimeric Bet v 1 during WT protein preparation.** *a*, separation of monomeric and dimeric Bet v 1 via ion exchange (*top panel*, peak corresponding to dimer indicated by *black lines*) and size exclusion chromatography (*bottom panel*). *b*, analysis of size exclusion chromatography fractions via native (*top panel*) and SDS (*middle* and *bottom panels*) PAGE. Comparison of reducing (*middle panel*) and nonreducing (*bottom panel*) SDS-PAGE demonstrates the reduction sensitivity of the dimer. *c*, SDS- and clear native-PAGE revealed stability of the correctly folded dimer under reducing conditions (*bottom panel*, *right side*). The bands corresponding to dimeric Bet v 1 are *boxed. Lane M*, marker; *lanes 17* and *35*, molecular mass of the marker band in kDa.

During purification, we observed WT Bet v 1a to elute as a double peak in ion exchange chromatography. Although the dominant peak (∼95% in all analyzed batches) was monomeric wild type Bet v 1a (as evidenced by MS analysis), the high salt fraction contained both monomeric and dimeric Bet v 1a, as judged by size exclusion chromatography (Fig. 1*a*). This interpretation was confirmed by clear native PAGE and SDS-PAGE (Fig. 1*b*). The SDS-PAGE analysis also revealed the Bet v 1a dimer to be reduction-sensitive. This observation was surprising, given the lack of cysteine residues in Bet v 1a. More intriguingly, the redox sensitivity of the Bet v 1a dimer can be further distinguished: one dimer species is completely redox stable under native PAGE conditions, whereas a second dimer species dissociates under reducing native PAGE conditions (Fig. 1*c*). We conclude that the Bet v 1a bridging is inherently redox-sensitive; depending on the quaternary structure, the bridging can be protected from reducing agents by the native dimer structure.

##### Crystal Structures of Dimeric Bet v 1a

**FIGURE 2.**
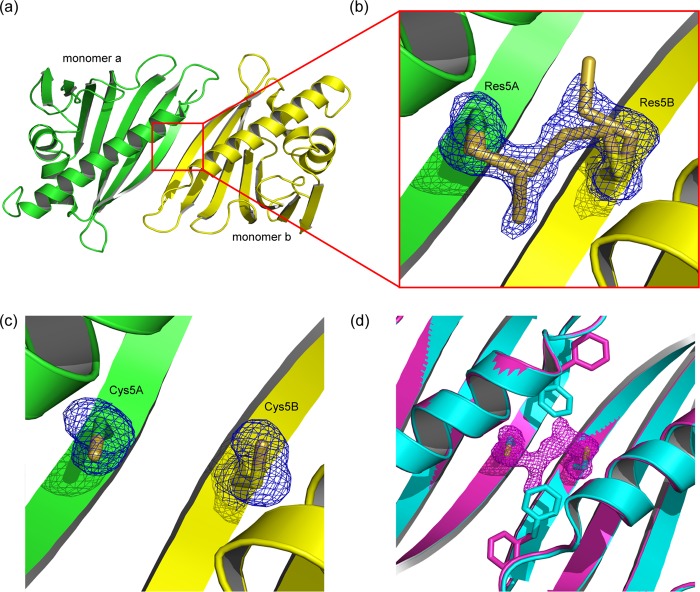
**Tetrasulfide dimer interface in the absence and presence of reducing agent.** *a*, cartoon representation of the assembly in the tetrasulfide dimer. The β1-strands of the monomers (*green* and *yellow*) elongate the antiparallel β-sheet over the whole quaternary structure. *b*, close-up view of the dimer interface. The tetrasulfide bridge exhibits partial breaks between Sγ_A_-Sδ_A_ and Sδ_B_-Sγ_B_. *c*, the dimer interface after reduction, with the linker electron density vanished. The residual electron density at residue 5 fits cysteines. *d*, comparison of Phe^3^ rotamers in the intact (*magenta*) and reduced (*cyan*) tetrasulfide dimer. Reduction leads to an approximately 120° flip of Phe^3^ toward residue 5. The presence of the linker (indicated by its density) keeps Phe^3^ in the same conformation as observed in the WT structure.

To elucidate the mechanism of the intriguing redox sensitivity of the Bet v 1a dimer, we crystallized dimeric Bet v 1a in different crystal forms at resolutions ranging from 1.17 to 1.73 Å. The overall fold of the monomers within the dimer structures was similar to monomeric WT Bet v 1a (*e.g.*, Protein Data Bank accession code 4A88 (18)). Although we found variations in the Bet v 1 dimerization mode, sheet extension was a unifying principle (Fig. 2*a*). The hereby primarily engaged β1 strands were slightly displaced as compared with monomeric structures. There were some minor differences in the backbone conformation of several loops, *e.g.*, the glycine-rich loop connecting β2 and β3 or the loop between β7 and the C-terminal α-helix. The statistics of data collection and model refinement are summarized in Table 1.

**TABLE 1:** **X-ray data collection and refinement statistics** The highest resolution shell values are shown in parentheses.

Data set	Tetrasulfide dimer	Tetrasulfide dimer reduced	Nonasulfide dimer	Bet v 1 Y5F
**Accession number**	4BKC	4BK6	4BKD	4BK7
**Data collection**				
Space group	P2_1_	P2_1_	C2	P2_1_
Cell dimensions				
*a*, *b*, *c* (Å)	39.9, 61.0, 59.7	40.1, 61.2 60.1	112.9, 44.7, 32.0	32.7, 55.8, 38.1
α, β, γ	90, 107, 90	90, 107, 90	90, 91, 90	90, 93, 90
Wavelength (Å)	0.8865	0.8865	0.8865	0.8865
Number of unique reflections	27,922	34,452	52,883	47,976
Resolution (Å)	41.62-1.73	57.33-1.63	56.45-1.17	55.81-1.14
*R*_merge_ (%)	0.119 (0.634)	0.062 (0.615)	0.027 (0.594)	0.035 (0.268)
Completeness (%)	98.1 (98.0)	99.5 (99.0)	97.8 (91.4)	96.3 (80.7)
Redundancy	3.0 (3.0)	3.4 (3.5)	3.2 (2.8)	3.2 (2.4)
*I*/σ(*I*)	5.7 (1.1)	13.2 (1.9)	17.0 (2.0)	17.1 (3.5)
Wilson B-factor	19.5	20.6	13.5	9.4

**Refinement statistics**				
Resolution range (Å)	56.97–1.73	57.33–1.63	56.45–1.17	38.04–1.14
Number of unique reflections	26,644	32,699	50,194	45,515
*R*_work_/*R*_free_ (%)	20.12/24.67	18.1/23.8	17.7/20.2	13.4/17.5
Number of atoms				
Protein	2,533	2,548	1,319	1,403
Water	219	428	198	209
RMSD from ideal values				
Bond lengths (Å)	0.005	0.004	0.008	0.012
Bond angles (°)	0.906	0.930	1.431	1.547
Average B-factors (Å^2^)				
Protein	19.56	19.54	17.07	12.47
Solvent	24.60	29.94	27.59	25.94

##### Covalent Dimerization

In a first crystal form (further referred to as “tetrasulfide dimer” as will be explained later), we found a Bet v 1 homodimer in the crystallographic asymmetric unit; the dimer was generated from a noncrystallographic, 2-fold symmetry axis in the center of the dimer interface (Fig. 2*a*). The dimer interface was formed by the N-terminal β-strands (β1) that were arranged in an antiparallel manner, and covered an accessible surface area of 536 Å^2^ per monomer (calculated with PISA (29)). The resulting 14-stranded β-sheet twists around the antiparallel C-terminal helices, which lie on the same side of the β-sheet. The dimer interface is stabilized by 13 hydrogen bonds and a symmetric salt bridge engaging Glu^127^ and Lys^137^ of either Bet v 1 monomer.

##### Covalent, Redox-sensitive Linkage at Residue 5

We identified residue 5, located at the symmetry axis, to play a unique role in the formation of the dimeric structure. Residues 5A and 5B are facing each other, with a distance of 4.75 Å between the Cα atoms. Intriguingly, the electron density at residue 5 was found inconsistent with the genetically encoded tyrosine. By contrast, the electron density indicates a covalent bridging between residues 5A and 5B.

We hypothesized that this linker density is related to the observed redox dependence of the dimer (Figs. 1, *b* and c, and 2*b*). To test this hypothesis, we soaked crystals with β-mercaptoethanol and subsequently determined the crystal structure at a resolution of 1.63 Å. Residues 5A and 5B were no longer connected, but the linker electron density apparently vanished upon reduction. The resulting electron density at position 5 could be consistently interpreted as cysteine (Fig. 2*c*). The reduction was accompanied by a reorientation of the neighboring Phe^3^ side chains in both monomers for ∼170°, now partly occupying the position of the phenol rings of tyrosine observed in WT Bet v 1 (Fig. 2*d*).

##### Bet v 1a Y5C Mutant Is Able to Reproduce Dimeric Bet v 1a

To confirm our electron density interpretation of residue 5 being a cysteine, we designed a Bet v 1a Y5C mutant to test whether a cysteine introduced at the dimer interface would lead to spontaneous dimerization. Analysis of purified Bet v 1a Y5C showed almost 90% dimerization and similar redox sensitivity as described before. Intriguingly, crystallization trials with Bet v 1a Y5C using the conditions identified for the tetrasulfide Bet v 1 dimer resulted in an unexpectedly low success rate, despite the high apparent homogeneity of the protein samples.

##### Mass Confirms WT Bet v 1a to Contain a Partial Y5C Exchange

To complement the results from the crystal structures and to get a detailed picture of the composition of the covalent linkage between the monomers, we performed mass spectrometry. We tested both dimers obtained from WT Bet v 1a, as well as Bet v 1a Y5C mutant preparation. In both samples, we detected intact masses of 34,822 Da and 17,411 Da. After reduction, both proteins had a mass of 17,380 Da, matching the expected mass of Bet v 1a Y5C. This observation implied that the covalently linked WT Bet v 1a does not contain the genetically encoded Tyr^5^. Instead, we concluded that cysteine should be present at position 5 with redox-sensitive adducts.

To further corroborate this conclusion, we reduced and alkylated both dimer preparations using iodoacetamide. The samples showed the expected mass shift of 57 Da resulting in 17,437 Da. In addition, tryptic digestion followed by sequencing of the obtained peptides identified the N-terminal sequence ^1^GVFNCETET^9^. Thus, we could unambiguously prove that dimeric Bet v 1 purified from the WT Bet v 1a preparation contains cysteine at position 5.

To elucidate the identity of the Cys^5^–Cys^5^ bridge, we tryptically digested a Bet v 1a dimer. The dimeric T_1–17_ peptide, containing Gly^1^–Arg^17^, could be chromatographically separated and identified using MS/MS. The T_1–17_ dimer peak could be further separated into three fractions, with an average monoisotopic mass shift between the three signals of 31.97 Da, representing a mass deviation of 0.63 ppm relative to the theoretical monoisotopic mass of elemental sulfur. The masses of the three species correspond to a disulfide-, trisulfide-, and tetrasulfide-linked dimer, respectively. The interpreted dimer mass (3,585.70 Da) matched the theoretical T_1–17_ dimer mass with an accuracy of 0.03 ppm. Furthermore fragmentation experiments not only confirmed the identity of the T_1–17_ peptide but also detected bond breaks within the polysulfide linker, further confirming the nature of the linker as di-, tri-, and tetrasulfide bridge.

##### Origin of the Y5C Exchange in WT Protein: Expansion of the Genetic Code

We followed two routes that could explain this exchange. First, we checked for trace contamination with a second plasmid. For this, we transformed *E. coli* BL21(DE3) with the resequenced Bet v 1a plasmid. Three isolated clones were used to produce WT Bet v 1a protein, confirming the spontaneous dimerization.

Second, we tested whether the Tyr codon UAC was site specifically misread to cysteine (UGC) and consequently affected by the expression temperature. Indeed, expression of Bet v 1a at 16 °C resulted in trace amounts of Bet v 1 dimer, compared with expression at 37 °C. Thus, temperature appears to be directly correlated with Bet v 1 Y5C incorporation. These observations suggest a context- and temperature-dependent “misreading” of the tyrosine triplet rather than a plasmid contamination.

We further investigated the mechanism of post-translational modification of Cys^5^. We identified the dialysis tubings as the main sulfur source, because they contain up to 0.1% sulfur in addition to several heavy metals. By extensive boiling of the dialysis tubing (more than 10-fold exchange), dimerization could be mostly prevented.

**FIGURE 3.**
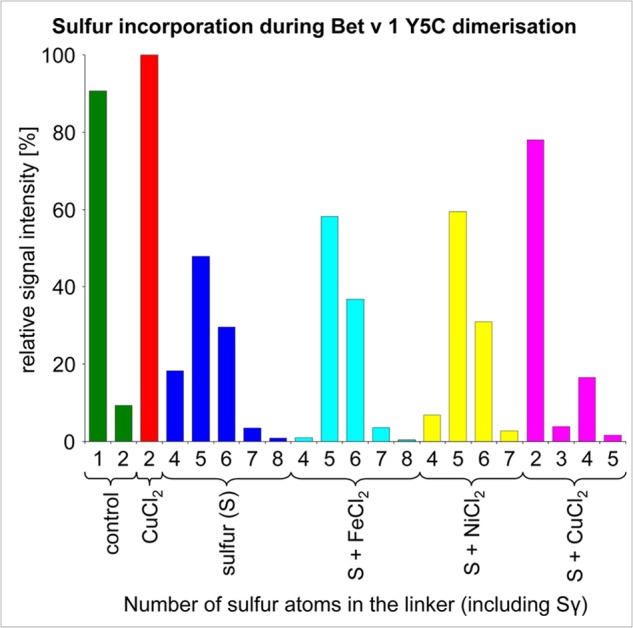
**Variation of sulfur atom incorporations in Bet v 1 Y5C.** The length of the linkers is dependent on the dimerization protocol. CuCl_2_ favors disulfide formation, reducing the amount of incorporated sulfur. By contrast, the addition of NiCl_2_ and FeCl_2_ had only a little influence on the polysulfide pattern.

Detailed analysis of the contaminations present in the dialysis tubings revealed that the dimerization could be reconstituted by the addition of CuCl_2_, whereby the addition of sulfur resulted in polysulfide rather than disulfide bridging. The addition of only elemental sulfur resulted in heterogeneous polysulfide linkage (Fig. 3). These results suggested that the contamination of the dialysis tubings, in particular sulfur and copper, triggered the observed dimerization of Bet v 1a Y5C.

##### Structural Diversity

Next to variations in the length of polysulfide bridges, several results indicated a remarkable heterogeneity of dimerized Bet v 1, *e.g.*, the blurred electron density of the polysulfide linker in the Bet v 1 crystal structures or the poor reproducibility of Bet v 1 dimer crystals with Y5C mutant preparations.

##### Tetrasulfide Linker Is Present in Open and Closed Configurations

After careful refinement, we could identify differences in the linker within individual data sets. In general, we observed breaks in the polysulfide linker (Sγ_A_-Sδ_A_-Sδ_B_-Sγ_B_) between Sγ_A_-Sδ_A_ and Sδ_B_-Sγ_B_ but apparently not between Sδ_A_-Sδ_B_ (Fig. 2*b*). Presumably, the sulfide breaks occur upon photo reduction upon x-ray exposure in the high intensity x-ray beam (30). Polysulfide reduction within the crystals is reflected by different Phe^3^ rotamers, partly occupying (albeit very rarely) the conformation as seen in the reduced crystal. This observation is in accordance with the MS fragmentation experiments, also showing breaks of the tetrasulfide bridge.

##### The Quaternary Structure of the Bet v 1 Dimer Depends on the Polysulfide Linker Length

**FIGURE 4.**
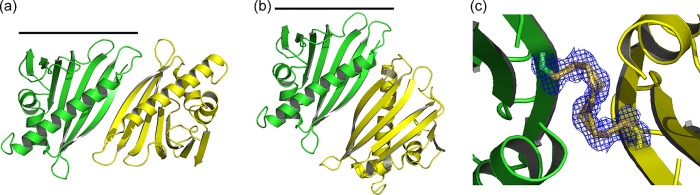
**Comparison of the quaternary arrangement in tetra- and nonasulfide bridged Bet v 1.** *a*, the tetrasulfide bridged dimer is stabilized by anti-parallel β-sheet formation, with an interface of 536 Å^2^. *b*, the nonasulfide bridged Bet v 1 is rotated along the sulfide bridge for ∼135°, resulting in a larger interface (670 Å^2^) and the loss of the intermolecular β-sheet extension. *c*, all nine sulfur atoms are resolved in the 1.17 Å resolution electron density.

We could crystallize a nonasulfide linked Bet v 1 dimer. These crystals belong to an alternative space group (C2) and diffracted to a resolution of 1.17 Å. In this nonasulfide structure, the dimer axis coincides with the crystallographic 2-fold symmetry axis, with the central sulfur on a (crystallographically) special position. The packing resulted in one molecule per asymmetric unit. Although both the tetra- and nonasulfide linked dimers exploit the N-terminal strand β1, their quaternary arrangement differs considerably, as evident by the C-terminal helix (Fig. 4). Most strikingly, the nonasulfide linker connected the distance of more than 12 Å between the cysteine C_α_ (Fig. 4*c*). As in the tetrasulfide dimer, alternative conformations of the linker could be detected.

Moreover, the quaternary structure can differ even in the presence of a disulfide linker, as indicated by different susceptibility to reduction. The reduction-insensitive dimer species was shown by MS to contain a classical disulfide bond (Fig. 1*c*). By contrast, CuCl_2_-induced disulfide dimer species were reduction-sensitive. This difference most likely reflects a difference in the quaternary structure, resulting in different solvent accessibilities of the disulfide.

##### Structural Relevance of the Dimer Assemblies

We hypothesized that the observed quaternary arrangement in the tetrasulfide and possibly also in the nonasulfide dimer structures reflects the (transient) dimerization that was observed with WT Bet v 1a (Tyr^5^) (11, 13). The proposed relevance of the dimer contacts would suggest that similar quaternary arrangements should be present in structurally related proteins. Therefore, we performed homology searches within the protein database using TopMatch (26).

##### Homology to the Tetrasulfide Dimer

**FIGURE 5.**
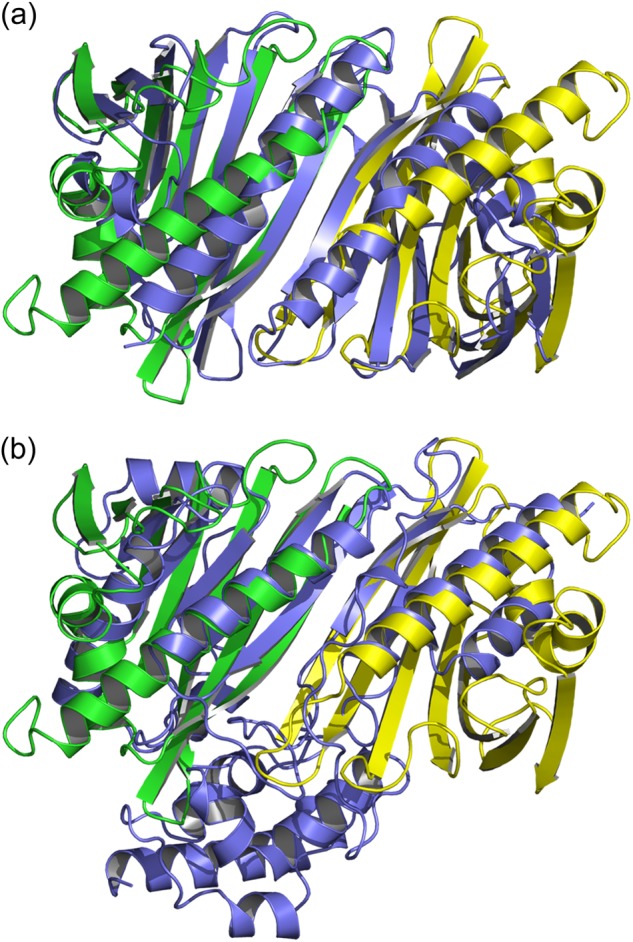
**Structural homologues with folds equivalent to the tetrasulfide dimer.** Bet v 1 is shown in *green* and *yellow*, and the homologues are in *slate blue. a*, SMU.440 from *S. mutans* forms stable homodimers in solution. *b*, the single chain arginine kinase from *L. polyphemus* exhibits an antiparallel β-sheet capped by two long helices, similar to the elongated sheet in the tetrasulfide dimer.

We identified several structures sharing the same overall fold as the tetrasulfide dimer. The closest resemblance was found with the protein SMU.440 from *Streptococcus mutans* (Protein Data Bank accession code 3ijt), (*a*) exhibiting a fold similar to Bet v 1 and (*b*) assembling in the same quaternary structure (Fig. 5*a*) (31). However, the structural architecture could be identified in several other examples, including the single-chain protein arginine kinase from *Limulus polyphemus* (Protein Data Bank accession code 3m10) (32) (Fig. 5*b*) and the crystal packing of abscisic acid receptor PYR1 monomers from *Arabidopsis thaliana* (Protein Data Bank accession code 3k3k) (33).

##### Nonasulfide Dimer Homology

Assemblies similar to the nonasulfide dimer interface were only found in the crystal lattice of several Bet v 1 variants (*e.g.*, Protein Data Bank accession code 1bv1 (34)). In addition, Hyp-1 from St. John's wort (Protein Data Bank accession code 3ie5) (35) exhibits a related arrangement. All identified nonasulfide-like dimer arrangements were built from crystal symmetry. These findings underline the inherent propensity of the Bet v 1-like proteins for quaternary arrangements primarily resembling that of the tetrasulfide dimer as observed in our crystal structures.

##### Influence of Bet v 1 Dimerization on Ligand Binding

**FIGURE 6.**
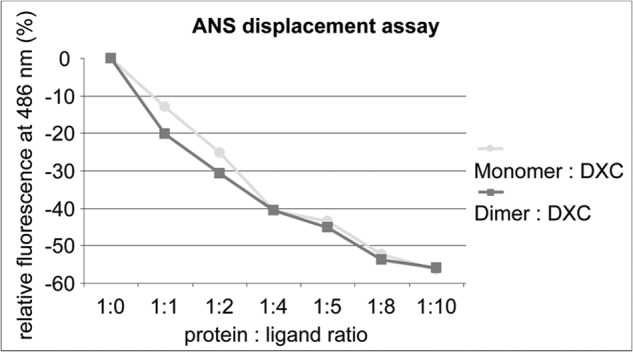
**Identical ligand binding capacity of monomeric and dimeric Bet v 1.** Changes in the fluorescence signal (Δ*F*/*F*_0_) of ANS induced by the presence of DXC in different molar ratios. Titration of both the monomer (*circles*) and the dimer (*squares*) gave comparable results for both ANS binding (ratio 1:0) as well as ANS replacement.

The ANS assay allows detection of ligand binding to Bet v 1. It is based on the increasing fluorescence signal of ANS at 474 nm when it is binding to hydrophobic patches (36). Displacement of ANS out of the hydrophobic core of Bet v 1 by a ligand results in a loss of the signal. The crystal structures of dimeric Bet v 1a revealed that the openings to the hydrophobic cavity are not covered by the interface, thus allowing ligand binding. For testing this structural interpretation, we chose the steroid DXC as a well characterized model substance (18, 37). As expected, DXC binding to dimeric Bet v 1a resulted in a decrease of ANS fluorescence at 474 nm, comparable to monomeric Bet v 1a, indicating displacement of ANS (Fig. 6). Consequently, dimeric Bet v 1a has ANS and DXC binding properties comparable to those of the monomeric form.

##### Immunological Relevance: IgE Binding to Bet v 1a WT/Y5F/Y5C

**FIGURE 7.**
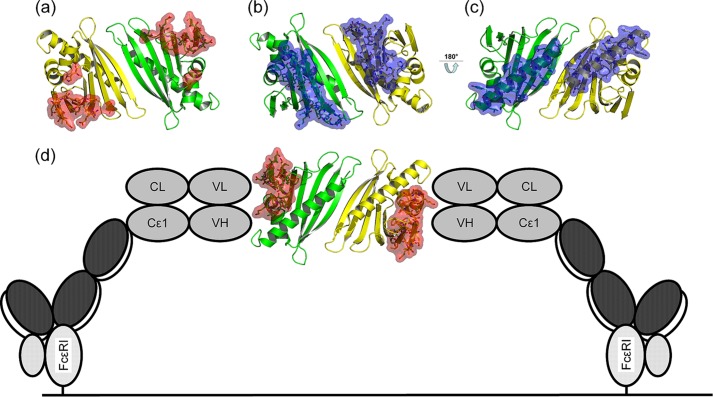
**Accessibility of IgE epitopes in the tetrasulfide dimer.** Residues corresponding to IgE epitopes are highlighted as sticks and surface. *a*, *b*, and *d*, three of four reported epitopes allow cross-linking of univalent IgE antibodies via dimeric Bet v 1 (shown as *red* surface) (38, 40). *c*, by contrast, a fourth epitope at the C-terminal α-helix of Bet v 1 (*blue* surface) is partly buried by the dimer interface, restricting simultaneous binding of two IgE (39). *d*, a schematic model for IgE cross-linking facilitated by dimeric Bet v 1 on the surface of an effector cell, mediated by FcϵRI, based on Ref. 7.

Cross-linking of FcϵRI receptors on the surface of effector cells is a hallmark event in the manifestation of allergic reactions. Analysis of four previously described Bet v 1 epitopes revealed that dimerization of Bet v 1 should allow largely unhindered cross-linking of univalent IgE antibodies via these epitopes (38–40) (Fig. 7). *In vitro* examination of IgE binding and cross-linking capacity of dimeric Bet v 1 was performed via ELISA and mediator release assay, using sera from patients allergic to Bet v 1. Next to WT Bet v 1a as standard, we tested dimeric Bet v 1a Y5C purified from the WT and mutant preparation and three monomeric Bet v 1a variants: Y5F, a true atomic mutation model to WT Bet v 1a, as revealed by its crystal structure (Protein Data Bank accession code 4BK7); reduced Bet v 1a Y5C; and a constitutively monomeric Bet v 1a Y5C variant whereby the Cys^5^ was disulfide-capped with glutathione (further referred to as Y5C^glu^), preventing dimer formation by blockage of the dimer interface.

##### ELISA Monomeric Variants

**FIGURE 8.**
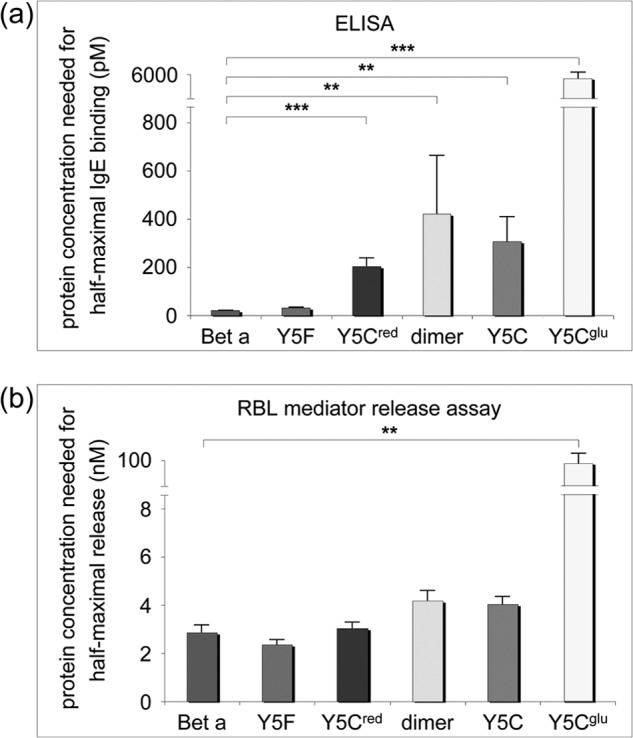
**IgE binding to monomeric and dimeric Bet v 1a variants tested via ELISA and rat basophile leukemia mediator release assay.** *a*, ELISA. The amount of protein needed for half-maximal antibody binding is plotted. Reduction of IgE reactivity correlated with alteration of the surface patch near residue 5. The strongest effect was obtained with Y5C^glu^. *b*, rat basophile leukemia (*RBL*) mediator release assay. The amount of protein that is needed for half-maximal mediator release (expressed as a percentage of total enzyme content in the cells) is plotted. No significant differences between monomeric and dimeric Bet v 1a variants could be detected. Y5C^glu^ induced a significant decrease in mediator release. *Bet a*, WT Bet v 1a; *Y5C^red^*, monomeric Bet v 1a Y5C (reduced); *dimer*, dimeric protein obtained from WT Bet v 1a preparation; *Y5C*, dimeric Bet v 1a obtained from mutant preparation; *Y5C^glu^*, Bet v 1a Y5C with Cys5 oxidized, and capped, with glutathione.

**FIGURE 9.**
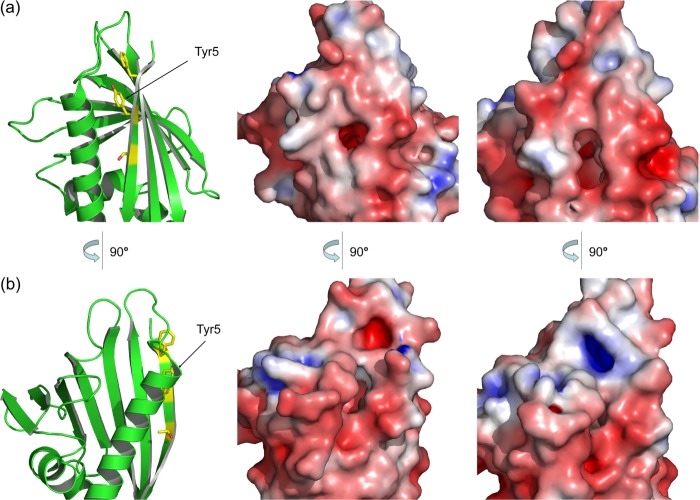
**Surface changes induced by the Y5C mutation.** The Y5C exchange propagates to the conformation of neighboring amino acids (cf. Fig. 2*d*), generating pronounced changes in shape and charge distribution at its environment. *a* and *b*, comparison of Bet v 1a and Bet v 1a Y5C. Two views of the affected region are shown. *Left*, schematic representation of Bet v 1a for orientation. *Middle*, Bet v 1a. *Right*, Bet v 1a Y5C.

WT Bet v 1a exhibited the highest IgE binding affinity. The conservative residue exchange Y5F led to a slight, insignificant decrease of IgE binding (1.46-fold). However, Y5C exchange had a stronger effect (9.03-fold) (Fig. 8*a*). This can be explained by a more pronounced change in the protein structure, affecting the mutation site itself and the changes in side chain conformations of the flanking residues Phe^3^ and Thr^7^ (Figs. 9 and 2*d*). Because the variations in these protein structures are strictly localized to the immediate environment of residue 5, we concluded that Tyr^5^ is part of a newly identified IgE epitope and involved in antibody binding, which might be due to IgE sera from different patient pools in this and earlier studies. Consistent herewith, the mixed disulfide Y5C^glu^ is an even more drastic modification of residue 5 and resulted in the strongest decrease in IgE binding (263-fold reduction) (Fig. 8*a*).

##### Trend Reversal in Dimer Variants

Given the importance of the N-terminal β strand with the central Tyr^5^ for IgE binding, one should expect an even stronger reduction in IgE binding upon Bet v 1 dimerization, because this would be the most drastic disruption of the proposed IgE epitope near residue 5. Intriguingly, IgE binding to dimeric Bet v 1 was only moderately reduced as compared with WT Bet v 1 (18.8- and 13.7-fold). We conclude that some compensatory IgE-binding site might be presented by the Bet v 1 dimer.

##### Rat Basophile Leukemia Cell Mediator Release Assay

Mediator release depends on antibody cross-linking on the mediator cells, which again directly depends on the IgE binding affinity in a complex way. Nevertheless, to a first approximation, the outcome of the mediator release approach should qualitatively reflect the results from ELISA experiments. Three monomeric Bet v 1a variants (WT, Y5F, and Y5C reduced) triggered a virtually identical release, as did the dimeric variants, despite their significantly reduced IgE binding. This compensation might be related to an improved cross-linking capacity of the dimer variants. By contrast, the mixed disulfide Bet v 1 variant Y5C^glu^ exhibited an almost 30-fold decrease in mediator release (Fig. 8*b*), in accordance with its reduced dimerization propensity and low IgE binding activity.

##### Immunological Relevance: Priming of the Immune Response

**FIGURE 10.**
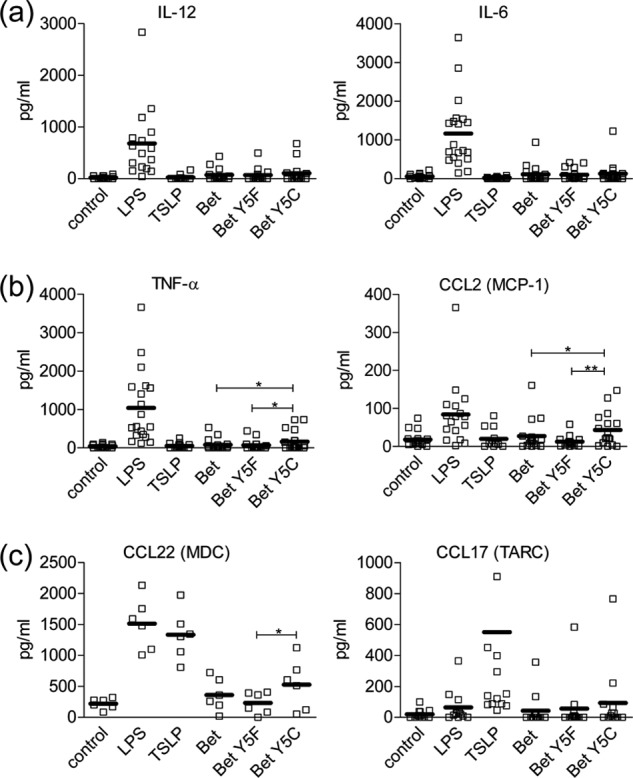
**Different Bet v 1 variants induce distinct cytokine releases in primary dendritic cells.** Cytokine secretion after stimulation of human primary DCs with 50 μg/ml of native Bet v 1a, Bet Y5F, or Bet A5C is shown. Uninduced cells (control) as well as cells stimulated with 10 ng/ml LPS and 30 ng/ml thymic stromal lymphopoietin were included as controls. Single values (*squares*) and the mean (*bars*) are depicted. Statistical significance was calculated with the Student's *t* test for each comparison of Bet v 1 *versus* Bet Y5F and Bet Y5C and for Bet Y5F *versus* Bet Y5C (*, *p* < 0.05; **, *p* < 0.01; ***, *p* < 0.005). *a*, T_H_1 priming cytokines IL-12 and IL-6. *b*, cytokines TFN-α and MCP-1, inducing a mixed polarization. *c*, T_H_2 priming cytokines MDC and TARC.

We further explored the immunological impact of dimeric Bet v 1a Y5C by stimulating primary DCs obtained from healthy donors. We used the same Bet v 1a variants as described for IgE binding studies, except Y5C^glu^. Given the high interpersonal variability with human samples, raw data of the cytokine releases were presented (Fig. 10). We used a panel of six cytokines that are related to different immune polarizations. Control experiments using the model stimulators lipopolysaccharide (T_H_1) and thymic stromal lymphopoietin (T_H_2) confirmed the established polarization type of IL-12 and IL-6 (T_H_1, T_H_17) (41, 42), TNF-α and MCP-1 (mixed polarization) (43, 44), and MDC and TARC (T_H_2) (45).

The three protein variants induced an indiscernible release of T_H_1-type cytokines IL-12 and IL-6 (Fig. 10*a*). Both TNF-α and MCP-1 secretion were enhanced upon stimulation with Bet v 1a Y5C as compared with Bet v 1a and Bet v 1a Y5F (*p* = 0.0275 for Bet v 1a Y5C *versus* Bet v 1a and *p* = 0.0304 for Bet v 1a Y5C *versus* Bet v 1 Y5F for TNF-α; *p* = 0.0494 for Bet v 1 Y5C *versus* Bet v 1a and *p* = 0.0062 for Bet v 1a Y5C *versus* Bet v 1a Y5F for MCP-1). Secretion of MDC was enhanced after stimulation with Bet v 1a Y5C as compared with Bet v 1a Y5F (*p* = 0.0360) and slightly enhanced as compared with Bet v 1a. TARC secretion was slightly enhanced after stimulation with Bet v 1a Y5C in comparison with Bet v 1a and Bet v 1a Y5F (in case of no *p* values given, the effect was too small).

## DISCUSSION

### 

#### 

##### The Quest for Bet v 1 Dimerization

Dimeric Bet v 1 variants from recombinant preparations were repeatedly reported in the literature. Most often, dimers were detected via SDS-PAGE or Western blot (11, 13–16) or by other biophysical methods including dynamic light scattering (13) or electrospray ionization Fourier transform ion cyclotron resonance MS (10). The underlying mechanism was, however, rarely touched. Schöll *et al.* (13) observed dimeric Bet v 1a under nonreducing conditions and showed dissociation to monomeric Bet v 1a upon incubation with glycerol at pH 6.2, suggesting a noncovalent dimer formation. Wellhausen *et al.* (46) detected dimeric Bet v 1a via Western blot under both reducing and nonreducing conditions.

##### Dimerization via Incorporation of Cysteine 5

We could identify the incorporation of cysteine at position 5 as one principal dimer-stabilizing mechanism in Bet v 1a. Intriguingly, the Cys5-mediated dimerization may result in different quaternary structures and sulfide bridges, including redox-sensitive and redox-insensitive disulfide and polysulfide forms. This observed propensity for dimerization reflects an underlying tendency for transient, noncovalent dimerization that is mediated by the N-terminal strand β1. These transient dimers are covalently cross-linked by the Cys^5^ incorporation by diverse mechanisms, as shown in this work.

##### Position-specific Alteration of the Genetic Code

The origin of the cysteine incorporation was puzzling. The most straightforward explanation appeared to be by mixed plasmids, which could be excluded by resequencing, followed by replication of cell transformation. Subsequent protein purification repeatedly (>5 times) reproduced the identified dimeric Bet v 1; hence the cysteine incorporation into wild type Bet v 1. Consequently, the cysteine incorporation must result from a “misread” of the coding triplet during transcription or translation. Because misreading frequency usually increase with higher temperature, we tested for cysteine incorporation at different temperatures. As expected, cysteine incorporation was minimized at the lowest expression temperature of 16 °C, where trace amounts of dimeric Bet v 1 could only be detected via Western blot. The observed temperature dependence is inconsistent with the alternative mixed plasmid hypothesis.

Site-specific misreading of codons was reported (*e.g.* Phe to Leu exchange (47)); however, this affected the base on the third position of the triplet (“wobble base”). By contrast, the triplets encoding tyrosine (UAC or UAU) and cysteine (UGC and UGU) differ in the second base, making the current finding highly remarkable.

##### Dimerization via Polysulfide Formation

Polysulfide formation in proteins is a rarely reported phenomenon. So far, human SOD1 represented the only published protein crystal structure exhibiting a polysulfide bond (Protein Data Bank accession code 3k91) (48). However, the reaction mechanisms underlying this particular covalent interaction are not investigated in detail, and it is in most cases not clear whether the modification originates from protein expression or the purification progress. In case of Bet v 1a, the polysulfide incorporation can occur spontaneously with the presence of elemental sulfur. Intriguingly, Cu^2+^ suppressed the formation of higher polysulfides by catalyzing the competing disulfide formation reaction. Only tetrasulfides were significantly occupied in the presence of copper, consistent with its preferred quaternary structure (Fig. 3). The catalytic role of copper may be explained by an intermediate chelating function to two cysteines that subsequently enables a proximity-driven disulfide bond formation that is accompanied by the release of Cu^2+^. Interestingly, also variation of the pH from 7.4 to 5.8 did not significantly prevent polysulfide formation, despite cysteines being protonated in this pH range. The major limitation to polysulfide formation in Bet v 1 is thus the presence of elemental sulfur.

Similar to SOD1, we identified dialysis tubings used during purification as the source for the additional sulfur. The comparison with SOD1 further underlines that the intrinsic propensity for dimerization dictates the type of polysulfide linker. The linker must be compatible with transiently formed quaternary arrangements, which are conversely hyperstabilized by a compatible polysulfide linker. This reciprocal dependence is nicely illustrated by the crystal structures of the tetra- and nonasulfide-bridged Bet v 1 dimers.

##### IgE Binding and Cross-linking Potency

Crystallographically observed dimer structures generally reflect their assembly in solution. It was therefore natural to analyze how dimerization affects antibody binding. The IgE-Bet v 1 binding can be modeled by the crystal structure of an IgG Fab fragment bound to Bet v 1 (49). This complex illustrates how the crystallographically determined Bet v 1 dimerization cross-links univalent IgE antibodies and consequently triggers the mediator release from mast cells (Fig. 7*d*).

##### Priming of the Immune Response: Stimulation of Primary Dendritic Cells

Primary DCs are the first immune cells to encounter an allergen. The induced up-regulation of cytokines, facilitating either T_H_1 or T_H_2 polarization, decides on the nature of the immune response toward the allergen. Because we found a strong impact of mutations at residue 5 on IgE binding, we tested WT Bet v 1a Y5F and Y5C for their effect on cytokine release (Fig. 10). The differential induction of cytokine release is consistent with a preferential T_H_2 response of the Y5C mutant. This mutant stabilizes the dimeric form of Bet v 1, which should influence its uptake by the dendritic cells (12). Once endocytosed, the Y5C polysulfide bridge may be reduced and possibly become monomeric during proteolytic processing. Even in that case, the Y5C mutation may influence the lysosomal degradation pattern of the allergen, as evidenced by the substantial changes in the surface topology and charge distribution (Fig. 9). Consequently, the change in the cytokine release by Bet v 1 Y5C may result from two major factors: the allergen uptake and, subsequently, its proteolytic processing.

##### Conclusions: Bet v 1 “Hot Spot” Region Centered at Residue 5

The identification of the dimeric Bet v 1a variant Y5C clearly suggests the so far neglected importance of the N-terminal region of the protein, especially residue 5. This relevance relates to the intrinsic binding properties of the N-terminal strand β1 and to the noncanonical Y5C mutation, harbored by β1, that results in various modifications. The strand β1 not only impacts the Bet v 1 homodimerization as well as IgE recognition, but also its uptake and/or processing by primary dendritic cells, as presented by changes in the cytokine profile.

## Footnotes

2 The abbreviations used are:

ANS: 1-anilino-8-naphthalene sulfonate
DXC: deoxycholate.
